# Forced-Air Warming and Resistive Heating Devices. Updated Perspectives on Safety and Surgical Site Infections

**DOI:** 10.3389/fsurg.2018.00064

**Published:** 2018-11-21

**Authors:** Wiebke Ackermann, Qianqian Fan, Akarsh J. Parekh, Nicoleta Stoicea, John Ryan, Sergio D. Bergese

**Affiliations:** ^1^Department of Anesthesiology, Wexner Medical Center, The Ohio State University, Columbus, OH, United States; ^2^Department of Anesthesiology and Perioperative Medicine, Xijing Hospital, Fourth Military Medical University, Xi'an, China; ^3^Department of Orthopedics, Wexner Medical Center, The Ohio State University, Columbus, OH, United States; ^4^Department of Neurological Surgery, Wexner Medical Center, The Ohio State University, Columbus, OH, United States

**Keywords:** forced air warming, Bair hugger™ patient warming, resistive heating, Hotdog® patient warming, surgical site infections, perioperative hypothermia

## Abstract

**Introduction:** Perioperative hypothermia is one of the most common phenomena seen among surgical patients, leading to numerous adverse outcomes such as intraoperative blood loss, cardiac events, coagulopathy, increased hospital stay and associated costs. Forced air warming (FAW) and resistive heating (RH) are the two most commonly used and widely studied devices to prevent perioperative hypothermia. The effect of FAW on operating room laminar flow and surgical site infection is unclear and we initiated an extensive literature search in order to get a scientific insight of this aspect.

**Material and Methods:** The literature search was conducted using the Medline search engine, PubMed, Cochrane review, google scholar, and OSU library.

**Results:** Out of 92 Articles considered initially for review we selected a total of 73 relevant references. Currently there is no robust evidence to support that FAW can increase SSIs. In addition, both of the two warming devices present safety problems.

**Conclusion:** As unbiased independent reviewers, we advise clinicians to weigh the risks and benefits when using either one of these devices; no change in the current practice is necessary until further data emerges.

## Introduction

Perioperative hypothermia (PH), defined as a core temperature below 36°C, is one of the most common phenomena seen among surgical patients (1). Hypothermia during surgery can be generated by various factors such as: exposure to surgical environment, thermoregulatory dysfunction during general or regional anesthesia and medications. PH leads to intraoperative blood loss, cardiac events, coagulopathy, an increase in hospital stay and associated costs (1, 2). Even mild hypothermia (approximately 2°C below normal temperature) can triple the incidence of wound infection and prolong hospitalization by about 20% (2). Passive and active warming methods are used in the operating room (OR) to prevent the incidence of PH. Blanket use to provide insulation and radiant heat loss prevention (passive method), warming intravenous fluids, and patient warming devices (active methods) are well known (3).

Currently, a plethora of patient warming devices are utilized to reduce the incidence of intraoperative hypothermia among surgical patients (2, 3) (Table 1). We focus our review on two most commonly used and widely studied methods—forced air warming (FAW) and resistive heating (RH)—based on recent publications associated with their use (3).

**Table 1:** List of patient warming option.

**Device**	**Description**
Resistive heating	Conductive polymer fiber sheet that produces heat and warms the patient through conduction
Forced air warming	Air is sucked in from the surroundings and warmed using electric coils. The blower circulates the warm air through a blanket that warms the patient through convection
Warm Blankets	Cotton Blankets warmed in a temperature controlled incubator
Circulating water garment	Heat pump circulates warm water through a patient worn garment
Water mattresses	Thermostatically controlled water-filled mattress that warms patient through conduction
Heated gel mattress	Thermostatically controlled gel based mattress that warms patient through conduction
Electric blankets	Blanket with inbuilt heating device
Radiant heaters	Electric heater that employs infrared radiation
Exothermic pads	Releases warmth through exothermic heat released when pads are exposed to air
Heated humidifiers	Warms and moistens air that passes over a heated water reservoir

FAW system employs air convection to transfer heat, utilizing a heat generator with a temperature management unit, a blower to circulate heated air and a temperature control system equipped with various sensors (4–6). The air enters through a 0.2 μm rated intake filter (4–6). The blower unit is connected, via rubber hose, to a disposable perforated air blanket (6). The warm air passes through this blanket and helps maintain patient's surface temperature within a physiological range (6).

RH system converts electrical energy into heat and warms the patient through conduction (2). Effective warming is delivered by non-disposable carbon polymer fiber fabric strips (7, 8).

Recently, the debate between FAW and RH has intensified. There is a concern that FAW device can increase the incidence of surgical site infections by disrupting the OR laminar air flow, (9–12) and by transporting contaminants from the floor to the sterile surgical field (13). Laminar flow ventilation is described as an entire body of highly filtered air (>99.997%) within a designated space (operating room) moving with uniform velocity (0.3 to 0.5 m/s) in unidirectional downward parallel flow (2, 9). Laminar flow ventilation system reduces the number of contaminants in the OR air by generating a continuous flow of low bacteria containing air and sweeping the contaminants away from the surgical area (9, 14). Some authors stressed the importance to check the compatibility of the devices used in the operating room (OR) with the laminar airflow system (13). The heat from FAW devices creates hot air convection current and disrupts the OR laminar air flow with a potential compromise of surgical site sterility and increased incidence of infections (2, 9, 15, 16). McGovern argued that FAW devices do not disrupt the laminar flow and the effect of OR laminar flow on the incidence of surgical site infections is questionable (9). Recently, several cohort studies (2, 17, 18) reported no significant benefit of laminar air flow in reducing the incidence of surgical site infections.

Furthermore, the difference in warming efficacy of these two patient warming devices is debatable. Some studies (19, 20) conclude that FAW is more effective than RH. Others (21) consider the RH being more effective or report (8, 13, 15, 22) the same efficacy (Tables 2–4).

**Table 2:** Studies favoring the use of FAW.

**References**	**Study description**	**The goal of the study**	**Conclusions**
(6)	Clinical study of 30 patients undergoing non-cemented hip implants	Amount of air bacterial contamination present in the OR with and without FAW use	No significant increase in bacterial counts with the use of FAW.
(23)	A cohort study of 63 surgical departments in Germany	Compared the effect OR Laminar flow v. Turbulent flow on the incidence of SSI	Significant higher incidence of SSI by using laminar flow compared to turbulent flow.
(24)	A control study using one volunteer	Evaluating the impact of FAW on laminar flow.	FAW does not reduce operating room air quality during laminar flow ventilation.
(25)	A pilot study with six patients	Evaluating if blankets increase bacterial counts in setting of OR laminar-flow	FAW does not increase the number of colony forming units (CFU) at the operating site and activity outside the laminar flow does not influence CFU on the table.
(26)	A study using eight healthy male volunteers	To determine if the use of convective warming therapy increased the risk of wound contamination	FAW when appropriately applied, does not increase the risk for airborne bacterial wound contamination.
(19)	A clinical trial with 60 patients undergoing laparotomy	Compares the efficacy of FAW and RH in maintaining intraoperative body temperature	FAW is superior to RH in maintaining body temperature during laparotomy.
(20)	A clinical trial with 28 patients	Comparing patient rewarming rates using FAW and RH	FAW warms patient at twice the rate than RH.
(1)	Clinical trial with 160 patients undergoing non-emergency surgery	Comparison of RH and FAW to prevent inadvertent perioperative hypothermia	FAW is more effective than RH in preventing postoperative hypothermia.
(14)	Clinical trial with 80 patients undergoing minor orthopedic surgery	Comparing the effects of RH and FAW on bacterial accounts in the OR	The type of patient warming system had no significant influence on bacterial counts on any sampling site.
(27)	Meta-analysis	Effectiveness of FAW for prevention of perioperative hypothermia in surgical patients	There was no statistically significant difference between FAW and RH in preventing perioperative hypothermia. However, FAW allows for a flexible selection of appropriate warming sites and provides better thermal comfort than RH.

**Table 3:** Studies not favor use of FAW.

**References**	**Study description**	**The goal of the study**	**Conclusions**
(4)	An experiment evaluating FAW intake filters.	Evaluating the efficiency of FAW intake filter.	The efficiency of intake filters is subpar and leads to internal buildup of contaminants which can be emitted into sterile OR.
(15)	An experiment assessing OR with laminar flow.	Comparing the effect of FAW and RH on OR laminar flow.	FAW disrupts laminar flow and creates a temperature gradient directly above the patient.
(9)	An experiment studying FAW and a clinical trial with 1437 patients.	Compares the effect of FAW and RH on disrupting the laminar flow and incidence of SSI	FAW disrupts laminar flow ventilation and significantly increases SSI in patients undergoing arthroplasty surgeries.
(5)	A simulated study to test FAW intake filters.	Evaluating FAW intake filter and its role in increasing contaminant count.	The intake filters are not very efficient in filtering air. It emits build up contaminants from inside the blower to sterile OR. The contaminants did contain disease causing organisms.
(28)	An case report of an epidemic	Isolating the source of epidemic outbreak of *Acinetobacter baumannii*	The source of an MDRO outbreak was isolated from colonization of organism inside FAW and the ventilation system.
(16)	An experiment using simulated orthopedic surgeries and bubble counts	Comparing the effect of FAW and RH on disrupting laminar flow. Also assessed the effect of drape height in assisting FAW with laminar flow disruption	FAW does disrupt ventilation flow over surgical site. Significant increase in bubble counts compared to RH and control groups. The drape height is not significant in affecting the FAW convection currents.
(29)	A simulated OR using FAW	Evaluate whether FAW are a source of contaminants	Concluded that these FAW devices are a potential source of nosocomial infection and proposed some design changes.
(22)	A clinical trial with 60 patients undergoing total knee replacement	Comparing the efficacy of FAW and RH in maintaining intraoperative patient core temperatures.	The RH is as effective as FAW in maintaining intraoperative body temperatures.
(11)	A clinical trial with 120 patients undergoing total hip or total knee arthroplasty	Comparing the safety and efficacy of RH vs. FAW in total joint surgery	FAW and RH achieved similar results in maintaining the core temperature of patients undergoing total knee or hip arthroplasty. The potential for airflow disruption is present with the FAW device and does not exist with the RH device.

**Table 4:** Advantages and disadvantages of forced air warming and resistive heating devices.

	**Advantages**	**Disadvantages**
Forced air warming	Easily performed Do not require direct contact between the blanket and the patient's skin Blanket is disposable	Potential safety hazard Energy consumption Fan noise The amount of skin covered is dictated by the extent of the surgical field The potential for bacterial colonization Increase in OR temperature
Resistive heating	Easy to clean No operating noise Can be reused Can be initiated immediately after induction of anesthesia	Potential safety hazard Requires manpower and labor time to clean Less flexible to be adaptable to patients posture Creases and wrinkles in the resistive blanket may impair performance and safety. Theoretically, patients are less comfortable Requires direct skin contact

Recently, the concerned manufacturers have commissioned scientists to conduct independent studies and refute publications that criticized their sponsored devices. Hence we decided to perform a review of all the available published literature in order to get a scientific insight of this present controversy.

## Methods

The literature search was conducted through the United Stated National Library of Medicine using the MEDLINE search engine, Pubmed, Cochrane review, Google Scholar and OSU library. The following keywords were used alone and in combination: Forced air warming, Bair hugger, Resistive heating, Hotdog patient warming, Surgical site infections, and Perioperative hypothermia. Out of 92 articles considered initially for review, we selected a total of 73 relevant references.

## Results

### The efficacy of FAW and RH considering temperature regulation

Several clinical studies analyzed the efficacy of FAW and RH devices without unanimous results. Some studies favored the use of FAW, while others recommended RH for warming patients during surgeries. Recent data indicated that core and mean skin temperatures were comparable among patients undergoing surgeries using the RH and FAW devices, both being equally effective in preventing hypothermia (7, 8, 15, 17, 22, 27). A study published by Perl et al., considering a limited sample size, concluded that heat transfer in RH group was significantly greater than the FAW group (21). Leijtens et al. reported an incidence of hypothermia of 26–28% during intra-operative FAW utilization (30). In contrast, Leung et al. and John et al considered that FAW is more effective than RH in maintaining intra-operative patient temperature (1, 19). Roder et al. indicated that FAW increased the patient's core temperature at twice the rate compared to RH when tested on 28 patients (20).

### The effect of FAW on or laminar flow

Several studies have focused on the effect of FAW on OR laminar flow using multiple techniques (smoke, temperature probes, and neutral-buoyancy detergent bubbles) to access and visualize the airflow patterns altered by patient warming devices. Recently, new experiments (some of them conducted in a simulated OR environment) evaluated the effect of FAW devices on disruption of unidirectional airflow and concluded that the exhaust heat from the poorly insulated FAW blankets increased the air temperature at the surgical side of the drape by >5°C. In this situation the warm air rose up producing thermal convection currents transferring the floor level air to the surgical site (5, 9, 12, 15, 16). The authors concluded that FAW disrupts unidirectional laminar flow. These floor level contaminants consist of microbial laden dust particles, desquamated skin, fibers from OR clothing/drapes and respiratory droplets (4, 5). McGovern et al. showed that convection current activity was highest just above the surgical site, while Dasari et al. inferred (using bubbles in simulated environment) that the currents were widely dispersed throughout the OR (9, 15). In contrast, when smoke was used to visualize the air flow and static (but heated) mannequins mimic surgical personnel, FAW system did not create an upward draft nor interfere with the effective function of the laminar flow process (9). McGovern et al. and Belani et al. compared FAW with RH and found that RH released non-significant exhaust heat with minimal influence on the laminar flow (9, 16).

### The effect of FAW on particular/bacterial count in the air

The effect of FAW on contaminant counts in the sterile OR environment (12, 25, 26, 31) generated different opinions among different authors. Studies conducted by Albrecht et al. found an increased bacterial load in OR room with ultra clean ventilation (UCV) when FAW is used, and attributed this effect to the floor level location of FAW blowers and their intake, where most of the contaminants originated (4, 6, 10). The FAW blower directed these contaminants into the blanket and near the surgical site. Albrecht et al. also examined intake filter efficiency and reported a 93.8% retention efficacy for 0.2 μm 200908C filters and 61.3% for 0.2 μm 200708D filters (4). This inadequate filtration capacity is linked to internal build-up of microbial contamination in vast majority of these blowers. The air paths of the FAW blowers were swabbed, and the probes revealed that 92–100% of the blowers carried viable microorganisms, with the heaviest growths detected at the distal end of the hose. *S. aureus*, coagulase negative staphylococcus (CoNS), micrococci, mold and MRSA, were detected in these devices (4, 5). Similarly, Gjolaj et al. reported bacterial buildup in 12 out of 29 studied blowers (32). The study of Avidan et al. proved that 40% of the FAW warmers grew *S. aureus, S. epidermidis, Corynebacterium* sp., *Crytococcus albidus*, and *Aspergillus fumigatus* in the airstream of these devices (29). Reed et al. indicated that 74% of their FAW devices grew CoNS-the most common pathogen responsible for prosthetic joint infection (5). Moreover, a case report by Bernards et al. described the outbreak of *Acinetobacter baumannii* that colonized the respiratory tract of 29 patients. The source of this pathogen was isolated from FAW devices and the hospital's ventilation system. Replacing FAW intake filters and cleaning the ventilation systems halted the outbreak (28).

Several studies used particle count in air samples to assess the air quality in OR and concluded that FAW does not worsen air quality in laminar flow operating room (2, 14, 25, 26, 31). Cristina et al. found no statistically significant correlation between microbial loads and particle counts when assessing OR air quality (33). Furthermore, Avidan et al. reported that although 40% of the swabbed FAW devices grew *S. aureus, S. epidermidis, Corynebacterium* sp., *Cryptococcus albidus*, and *Aspergillus fumigatus*, the resultant air does not grow any of these aforementioned organisms (29).

### The effect of FAW on the rate of SSI

A limited number of clinical studies referred to the effect of FAW on SSI rates. An extensive retrospective/ simulated prospective study published by McGovern et al. in 2011, and involving 1,437 total joint arthroplasty patients, showed a significant increase in deep joint infection at the rate of 3.1% when FAW was used, compared to 0.8% when conductive heating was used (9). Nonetheless, the retrospective nature of data collection poses limitations when considering antibiotic regimens and some other non-standardized covariates. The authors isolated microbes from cultures, and found the ones responsible for septic joints—predominantly skin commensals—suggesting that the pathogens may be transported and deposited through air (9). Morreti et al. found a significantly increased bacterial load in the air samples when FAW was used, yet none of the patients developed nosocomial infections (6).

### Safety use of forced air warming and resistive heating system

Although these devices are widely used, they pose a significant burn risk to the patients. Several articles have reported FAW related burn injuries and pressure ulcers when placed improperly on patient's skin (2, 34–37). Mayo stands, trays and surgical equipment restrict the warming blanket from expanding and the non-circulating warm air increases the risk of burns (2). FAW misuse is one of the main causes of burn injuries (38). Similarly, RH was associated with thermal injuries, a case report published by Dewar et al.describing three pediatric patients who were diagnosed with thermal injuries, two of them requiring skin grafts and long term scar management (39).

## Discussion

There is a lot of discrepancy when comparing the efficacies of FAW and RH. This can be attributed to variation in study designs, type of surgeries, the anatomical location of the warming blankets (either anterior, posterior, upper body, or lower body), the difference temperature settings, and temperature measurement methods (1, 8).

Whether FAW increases the amount of particles in the air is controversial (12, 24). Recent studies have reported that the filter used in FAW devices is not very efficient (4). Reed et al. and Brown et al. suggested that replacing the FAW intake filter with a high efficiency particulate air (HEPA) filter and an additional filter at the outlet hose end will reduce contaminant counts (5, 40). Some authors advised to use FAW devices according to manufacturer's instructions in order to avoid bacterial contamination of the surgical field (29, 41, 42).

The efficacy of UCV laminar flow in the OR was questioned by different sources (2, 23, 43–45). A cohort study conducted by Gastmeier et al. questioned the laminar air systems benefit over conventional ventilation systems (43). Hooper et al. drew similar conclusions when studying lateral and vertical UCV in 51,485 total hip replacements and 36,826 knee replacement patients (44). Similarly, a large study conducted in Germany with 99,230 patients compared the incidence of SSI in hip prosthetic surgery using laminar ventilation vs. turbulent ventilation; the results showed a significant increase in SSI when laminar ventilation was involved, compared to turbulent ventilation (23). Similar conclusion was reached by another large scale rectrospective, data collection (0–14 years) from Norwegian Arthroplasty Registry conducted by Engesæter et al. involving 22,170 hip arthroplasties (45). Furthermore, studies have discussed the influence of drapes hanging between the surgical site and anesthesia on laminar flow. Vertical drapes can lead to production of accessory convection currents that disrupts laminar flow, and eliminating the vertical drapes will facilitate the release of exhaust heat without interrupting the laminar flow near the surgical field (12). Additional sources of heat in the OR, including surgical lights and personnel, play an important role in ventilation disruption (15).

Very few studies have directly researched the link between FAW and SSI with limitations. Mcgovern et al. conducted a study on 1,437 hip and knee arthroplasty surgeries and showed a significant increase in incidence of SSI using FAW (9). During the course of the study several changes in the prophylactic antibiotic and anti-thrombolytic regimen influenced the results. Moreover, multiple covariates associated with SSI such as high BMI, clinical factors (incontinence), and blood transfusions were not assessed/ documented.

Most of the studies did not mention whether the FAW devices were maintained and used according to manufacturer's instructions, whether they changed the filter every 6 months or 500 h, or whether they attached the hose to a new, manufacturer-approved warming gown for each patient. There are many methodological concerns regarding these studies related to sample size, randomization, blinding, and selected patient population in order to obtain unbiased results. Certain studies concluding that FAW warming devices do have a potential to increase particular or bacterial counts, were supported by manufacturing companies with competing interest.

A significant number of studies suggest that both FAW and RH are equally effective in maintaining core body temperatures and reducing hypothermia (7, 8, 15, 17, 22).

## Conclusion

The benefits of maintaining normothermia during the perioperative period is well documented. Many studies suggest that FAW can disrupt laminar flow system, and transport floor contaminants directly to the sterile surgical field, but the link between disrupted laminar flow, increased contaminant count, and SSI is unclear. More prospective, controlled, well designed, large scale clinical trials are warranted to compare these two different warming systems. As unbiased independent reviewers, we advise clinicians to weigh the risks and benefits when deciding to use either one of these devices. We advise to keep the current practice unchanged until further data emerges.

## Author contributions

WA Initial concept, actively involved in literature review, writing, editing/review of manuscript, final version of the manuscript, NS Initial concept, editing and review the final version of manuscript, SB Senior author involved in initial concept, manuscript review, and final version of the manuscript, JR Manuscript review, QF, AP literature review, writing of manuscript.

### Conflict of interest statement

The authors declare that the research was conducted in the absence of any commercial or financial relationships that could be construed as a potential conflict of interest.
